# Structural flexibility of 4,4′-methylene diphenyl diisocyanate (4,4′-MDI): evidence from first principles calculations

**DOI:** 10.1007/s00894-014-2097-8

**Published:** 2014-02-13

**Authors:** Pawel Rodziewicz, Jakub Goclon

**Affiliations:** 1 Institute of Chemistry, University of Bialystok, Hurtowa 1, 15-399 Bialystok, Poland; 2 Interdisciplinary Center for Molecular Materials (ICMM) and Computer-Chemistry-Center (CCC), Friedrich-Alexander-University Erlangen-Nürnberg, Nägelsbachstr. 25, 91052 Erlangen, Germany

**Keywords:** 4,4′-methylene diphenyl diisocyanate, 4,4′-MDI, C-H ⋯ *π* hydrogen bonds, Car-Parrinello molecular dynamics, Structural flexibility

## Abstract

A reactant used globally in the production of polyurethane is the molecule 4,4′-methylene diphenyl diisocyanate (4,4′-MDI). The structural flexibility of 4,4′-MDI is one of the most important molecular properties influencing the polymerization process and this property was therefore modeled using density functional theory (DFT) calculations and Car-Parrinello molecular dynamics (MD) simulations. Global and local minima structures were found and confirmed by vibrational analysis. The energy barriers related to rotation of the aromatic rings were estimated by DFT calculations. The stability of global and local minima was verified by Car-Parrinello (MD) runs at finite temperature. The presence of weak C–H⋯π hydrogen bonds was confirmed by atoms in molecules analysis and found to be responsible for the low energy barriers.

## Introduction

Polyurethanes are the products of the polyaddition reaction of diols and diisocyanates. This class of polymers has numerous applications in the automotive industry, usually as soft or rigid foams and flexible plastics [1]. The production of polyurethanes is thus one of the most important branches of the global chemical industry. In that context, it is worth mentioning that the isocyanates needed in the polyaddition reaction are highly toxic and hazardous reagents [2].

**Fig. 1:**
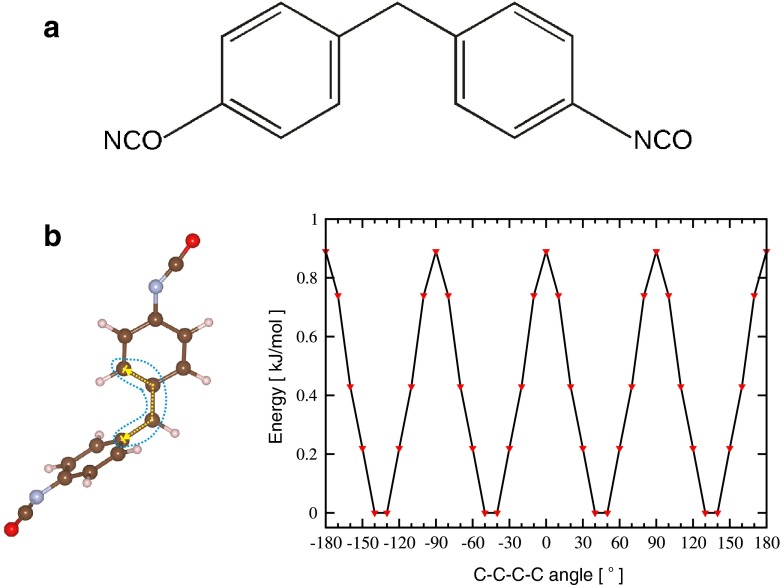
**a** Simplified structural formula of the methylene diphenyl diisocyanate (4,4′-MDI) molecule. **b** Optimized energies (kJ mol^−1^) of 4,4′-MDI as a function of the constrained value of the C–C–C–C dihedral angle (*right*). The angle of interest is marked with a* dashed line* (*left*). All calculations were performed at the density functional theory/Perdew-Burke-Ernzerhof (DFT/PBE) level, using Vanderbilt ultrasoft pseudopotentials, with plane-waves cutoff of 30 Ry

In this work, we focused on methylene diphenyl diisocyanate (4,4′-MDI, see the structural formula in the top panel in Fig. 1), which is one of the most frequently used diisocyanates in the production of polyurethanes [3]. The molecule of MDI can exist in three isomers, among them, the 4,4’-methylene diphenyl diisocyanate (4,4’-MDI) is prevalent in the chemical industry.

The polymerization reactions involved are very complex chemical processes that are strictly dependent on thermodynamic conditions. Even small changes in reaction conditions such as temperature or the concentration of reactants can significantly influence product formation. Physicochemical properties, e.g., structural flexibility, of the 4,4′-MDI molecule are also very important for chain formation during the polymerization process. The main aim of the industrial production of polyurethanes is to control precisely the physicochemical properties of the final product. The majority of studies on these polymerization reactions focus on experimental work. Theoretical models are difficult to construct due to the amount of reactant molecules involved in the polymerization reactions and the long time scale needed to represent the calculations. Theory, however, can significantly support experimental results, not only by reproducing the data obtained but also by explaining the chemical reactions on the molecular scale.

Molecular modelling is one of the techniques used in polymer reaction engineering. A variety of methods, from molecular mechanics to ab initio quantum calculations, is used to model chemical reactions, with the method of choice depending on system size [4, 5]. The most suitable approach, but also one of the more computationally expensive, is to model polymerization processes including the formation and breaking of new bonds using electronic structure-based methods like, for example, density functional theory (DFT).

Computational studies of 4,4′-MDI species to date have been limited to the so-called repeat units of 4,4′-MDI, which were used either as building blocks in the shape memory polyurethane model [6] or to investigate hydrogen bonds between hard–hard and hard–soft polymer segments [7]. The properties of polyurethane block copolymers emerge both from investigations of interactions between hard-segments and the conformation of the smallest building blocks.

Our modelling approach used DFT to study the structural and vibrational properties of the 4,4′-MDI molecule—the main reactant in the production of polyurethanes. A detailed study of all possible conformational changes of a single molecule is an important step in the understanding of the aggregation process of MDI molecules and also of polymerization itself.

Two phenyl rings in the 4,4′-MDI molecule are connected by the –CH_2_ group; therefore, their mutual rotation should be rather free of steric obstacles, unlike, for example, a biphenyl molecule [8]. Both phenyl rings cannot form one single plane so π – π stacking interactions are not present. The rotation of one phenyl ring might, however, cause the formation of a weak intramolecular C–H⋯π hydrogen bond between the hydrogen atom from the aromatic C–H bond and the region with the π electron acting as the proton acceptor. This class of weak H-bonds also has interesting spectroscopic properties, namely the C–H bond of the proton donor might be contracted and, as a result, the respective *ν* (C–H) stretching frequency is blue-shifted [9].

The formation of the intramolecular C–H⋯π hydrogen bond might, however, have important implications for the aggregation of 4,4′-MDI molecules. The interaction energies estimated for the π – π stacked dimers and systems with the C–H⋯π hydrogen bond are comparable [9, 10]. Molecule–molecule interactions driven by intermolecular π – π stacking have to compete with intramolecular C–H⋯π H-bonds. The π stacking interactions are well known phenomena in the analysis of acid–base pairs in DNA [11]. On the other hand, C–H⋯π H-bonds are known to be responsible for cooperative effects in cocrystals as the structure-determining factor [12, 13].

In this work, we studied the conformational flexibility of a single 4,4′-MDI molecule theoretically using combined static density functional theory (DFT) calculations and Car-Parrinello molecular dynamics (MD) simulations. Such information may serve as a basis for further modeling of 4,4′-MDI aggregation processes. The results obtained from such modeling may prove useful in the planning of synthesis strategies of new materials with desired properties. A detailed knowledge of the conformational flexibility of the 4,4′-MDI molecule might be helpful in the synthesis of new nanocomposite polyurethane materials with desired thermoplastic properties, as has been shown, for example, in the case of the mixture of MDI, polyols and organoclay [14]. Beside this, the spectroscopic properties obtained in our calculations might be used to understand experimental infra-red (IR) spectra since such data are very often used to control and recognize the hard-segments in polyurethane block copolymers [15].

## Computational methods

All DFT calculations used the gradient-corrected Perdew-Burke-Ernzerhof (PBE) exchange-correlation functional [16] performed using the CPMD program package [17]. Extensive test calculations showed that the PBE functional gives satisfactory precision for the description of hydrogen bonds [18]. To properly describe weak intramolecular electrostatic interactions, the empirical dispersion correction proposed by Grimme was used [19].

The electronic wave functions were expanded in a plane-wave basis set with a kinetic cutoff energy of 30 Ry, and the effective potential of ions was described by Vanderbilt ultrasoft pseudopotentials [20]. We used a cubic cell of side a = 25.0 Å with periodic boundary conditions so that the Brillouin zone sampling was restricted only to *Γ* -point. The Poisson equation for the isolated system was solved using Martyna and Tuckerman’s method [21]. Each fully optimized structure was checked as a stationary point by means of vibrational analysis.

Car-Parrinello (CP) MD [22] simulations were performed in the canonical ensemble (NVT) utilizing Nosé-Hoover chain thermostats [23, 24] for nuclear degrees of freedom. The temperature in the CP-MD simulations was set to 350 K, i.e., slightly higher than the melting point. As the initial atomic configuration for the CP-MD runs we took the optimized global minimum energy structure obtained in the DFT calculations. The production run for CP-MD simulations was 24 ps long and preceded by a stepwise 6 ps equilibrium procedure. The fictitious electronic mass was set to 400 a.u. and a time step of 4 a.u. was used.

Atoms in molecules (AIM) analysis was carried out using the AIMAll 13.05.06 program package [25]. For some structures taken as snapshots from the CP-MD trajectory, we performed full optimization of the wavefunction using the Gaussian 09 Rev. A.01 program package [26]. Here, we also used the PBE exchange-correlation functional with the Grimme dispersion correction and double zeta basis set.

All figures were produced using the program VESTA [27]. CP-MD runs were visualized with the help of the VMD program package [28].

## Results and discussion

### Global and local minima

The structural flexibility of the 4,4′-MDI molecule is correlated mainly to the presence of the –CH_2_ group between two phenyl rings substituted by isocyanate groups, which enables their independent rotation. The 4,4′-MDI molecule cannot be planar due to the presence of the –CH_2_ group, which imposes a fixed angular dependence between the planes represented by the two phenyl rings. The existence of the –CH_2_ group also has other implications, namely the hydrogen atom from the aromatic C–H bond from one phenyl ring can interact with the π electron density from the second phenyl ring. Thus, an intramolecular C–H⋯π hydrogen bond might be formed in the 4,4′-MDI molecule.

Static DFT calculations were performed to study the potential energy surface of the mutual phenyl rings rotation, with the special focus on the formation of weak intramolecular C–H ⋯ π H-bonds. The rotation of both phenyl rings with respect to the single C–C bond was modelled by the change of the selected C–C–C–C dihedral angle (see Fig. 1) every 10° to mimic full phenyl ring rotation.

Figure 1 shows the optimized energy values of local minima of the 4,4′-MDI molecule as a function of the constrained C–C–C–C dihedral angle, whereas the positions of all remaining atoms were optimized. The energy values do not refer the absolute energy in atomic units but, for transparency, these are given in kJ mol^−1^ unit with respect to the total energy of the lowest energy structure.

Different conformations of the 4,4′-MDI molecule arising from rotation of the phenyl rings may be considered as symmetrical in the range of the C–C–C–C dihedral angle values: −180 → 0 and 0 → 180. From a theoretical point of view, such symmetrical structures differ only with respect to the position of the isocyanate group. The isocyanate group is always in the same plane as the phenyl ring. Therefore, we performed calculations for 4,4′-MDI structure with the constrained C–C–C–C dihedral angle of 180° with two different positions of one isocyanate group in order to mimic the influence of its rotation on the total energy values. The energy difference between the optimized structures with two positions (left and right) of the NCO group was calculated to be only 0.02 kJ mol^−1^. Such low value for this energy difference is, however, not surprising taking into account the large distance between the isocyanate groups and their lack of mutual interaction.

The configurations of the 4,4′-MDI molecule are not only symmetrical for the C–C–C–C dihedral angle values of 0 and 180 degrees; they are also symmetrical when the C–C–C–C dihedral angle is equal to 90 or −90 degrees. This effect is due to rotation of the second, unconstrained phenyl ring, in the geometry optimization process. Thus, the C–H⋯π contact is preferred over the wing-like orientation of both phenyl rings.

Geometry optimization with constraints was the first step toward full evaluation of the potential energy surface of the 4,4′-MDI molecule. The plot shown in Fig. 1 suggests the existence of energy barriers with respect to the rotation of one phenyl ring. The highest values of the relative energy start at a C–C–C–C dihedral angle value of 0 and are repeated every 90°. The same interval of 90° holds for the lowest energy values. The energy difference between the highest and the lowest value is only 0.89 kJ mol^−1^ and suggests almost barrierless rotation of the phenyl rings with respect to the single –C–C– bond. It is worth noting that the structures obtained in the geometry optimization procedure with constraints cannot be considered as true local minima.

**Fig. 2:**
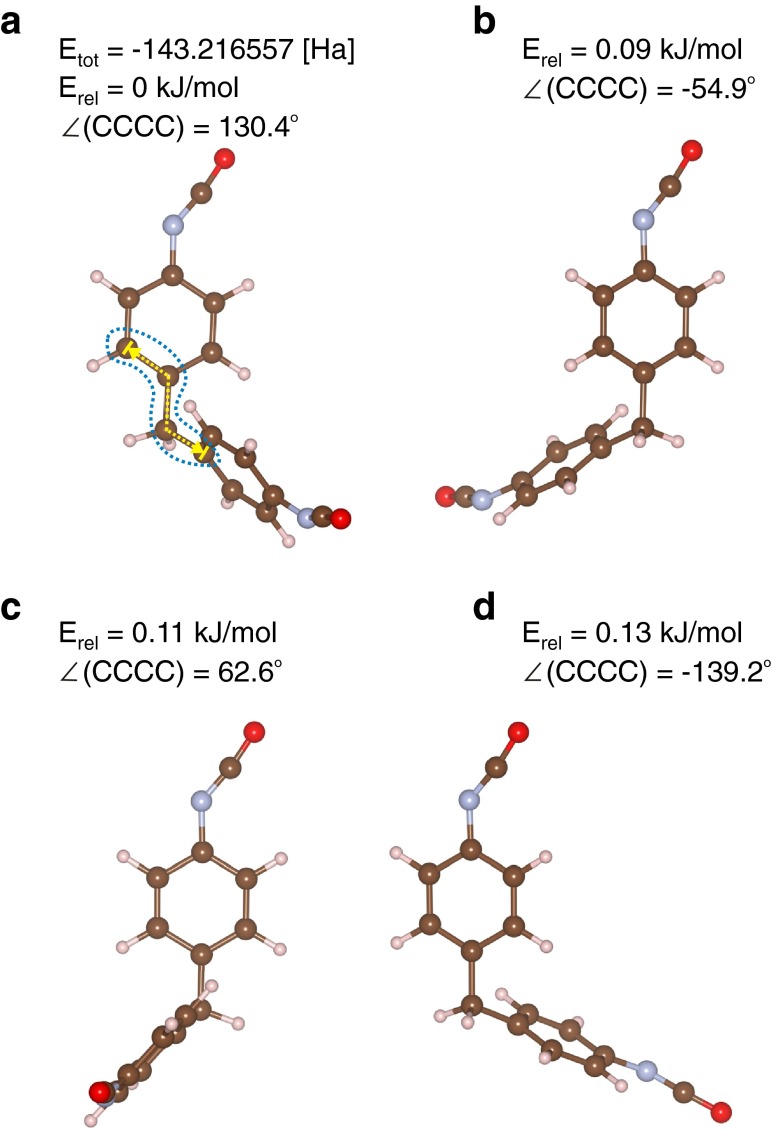
Optimized structures of 4,4′-MDI molecule without constraints (*A*–*D*). The absolute and relative energies are given in Hartree [Ha] and kJ mol^−1^, respectively, whereas the values of the C–C–C–C dihedral angle are given in degrees. All calculations were performed at the DFT/PBE level, using Vanderbilt ultrasoft pseudopotentials, with a plane-waves cutoff of 30 Ry

The second step of the calculations was to find all local minima utilizing the results shown in Fig. 1 as initial configurations and reoptimizating the geometry without any constraints. Figure 2 shows four structures that correspond to the minima shown in Fig. 1. All structures in Fig. 2 were verified as real stationary points by vibrational analysis. We labelled the structures as A–D in Fig. 2 and in principle could designate structure A as the global minimum and B, C and D as local minima. The C–C–C–C dihedral angle in the A–D local minima changes slightly with respect to the initial configurations optimized with constraints. The absolute value of the energy of structure A is given in Fig. 2. Other minima are ordered according to their relative energy calculated with respect to structure A.

It is worth mentioning that the absolute energy value of the global minimum A is, at the accuracy given, the same with respect to the lowest energy structure as that obtained in the geometry optimization with constraints. This is not surprising since the optimized C–C–C–C dihedral angle value differs only by 0.4°. Release of the C–C–C–C dihedral angle constraint in the geometry optimization procedure, however, lowers the absolute energy of other local minima. The values of the relative energies for the local minima A–D are significantly lower than 1 kcal mol^−1^ (4.18 kJ mol^−1^, the so-called “chemical accuracy”) so we consider all of them to be energetically equivalent. Although we report all energies with a precision of two decimal places to distinguish the structures, we are aware that this suggests an accuracy that is beyond the current calculations.

The structures shown in Fig. 2 are depicted in such a way that the top phenyl ring always has a fixed position. This visualization of all local minima structures enables similarities between them to be found by their superimposition. Indeed, a quick overview suggests the existence of an element of symmetry, namely two mirror planes. Both structures A and B might be horizontally mirrored to structures C and D. The only structural element that might influence this symmetry operation, and break it, is the isocyanate group. As mentioned before, the energy difference within the structures with a different NCO group position is negligibly small.

All local minima A–D shown in Fig. 2 have one common structural motif, namely a weak C–H⋯π hydrogen bond. The presence of the rigid –CH_2_ group between the phenyl rings enables such weak C–H⋯π H-bond formation only when the plane formed by one ring is perpendicular to the second. For all local minima (A–D), the two phenyl rings are not fully perpendicular to each other but are skewed so that two very weak C–H⋯π H-bonds are formed. Both phenyl rings are simultaneously proton donors and acceptors due to the presence of the aromatic C–H bond and π electron density, respectively. A simple measure of the strength of such C–H⋯π interactions is the distance from the hydrogen atom from the C–H group of one phenyl ring to the closest carbon atom from the second phenyl ring. The H⋯C distances for all local minima are collected in Table 1. In the case of structures A and B, the two H⋯C distances are almost the same so that both configurations are very symmetrical, whereas for other local minima C and D, one of the H⋯C distances is shorter and the other longer than the corresponding distances in the A and B structures. Furthermore, the average distance between the H atom and all six C atoms was calculated and listed in Table 1. The average H⋯C distance (<H⋯C> _avg_) shows the weakness of the hydrogen bond formation.

**Table 1:** Energy, structural and spectroscopic parameters of the global and local minima of 4,4′-methylene diphenyl diisocyanate (4,4′-MDI). Relative energies (*E*_rel_) are given kJ mol^−1^. Geometrical parameters: dihedral angles [∠ (CCCC)] and interatomic distances (H⋯C and <H⋯C> _avg_) are given in degrees and Ångstroms, respectively. The shifts in the vibrational frequency of C–H bonds [Δ ν (C–H)] in cm^−1^) engaged in the C–H⋯π H-bond were calculated with respect to the non-bonding C–H group. The corresponding changes in C–H bond length [Δ (C–H)] are also presented

Label	*E*_rel_ (kJ mol^−1^)	∠ (CCCC) (°)	H⋯C (Å)	<H⋯C>_avg_ (Å)	Δ (C-H) (Å)	Δ ν (C-H) (cm^−1^)
A	0	130.4	2.81	3.77	−0.0003	1.8
			2.81	3.77	−0.0003	1.8
B	0.09	−54.9	2.85	3.79	−0.0004	7.9
			2.88	3.83	−0.0001	0.5
C	0.11	62.6	2.72	3.59	−0.0003	2.3
			2.98	4.00	−0.0004	6.9
D	0.13	−139.2	2.68	3.53	−0.0005	3.4
			3.09	4.16	−0.0004	6.1

Another structural consequence of the formation of C–H⋯π H-bonds is the change in C–H bond length. In the 4,4′-MDI molecule, one C–H bond in the phenyl ring interacts with the π electron density from the second ring. In the case of intramolecular hydrogen bonds, the reference structure must be defined in order to estimate the change in bond length upon H-bond formation. As a model for the reference distance, the non-perturbed aromatic C–H bond was used. Since both the C–H bond involved in the interaction and the non-interacting C–H bond have the same chemical environment, the difference between them should be a good measure of the strength of the C–H⋯π H-bond. All the C–H bond length differences [Δ (C–H)] are presented in Table 1. The changes in C–H bond length upon interaction with the π electron density are quite small and do not exceed 0.0005 Å. Since the binding energy cannot be unambiguously calculated in the case of intramolecular H-bonds, one can correlate C–H bond changes with data on intermolecular H-bonds.

A common practice is to estimate the strength of the A –H⋯B –H bond via correlation with the shift in *ν* (A–H) stretching vibration [29]. For 4,4′-MDI, the difference between the *ν* C–H stretching vibration of the C–H bond involved in the C–H⋯π interaction and the non-interacting C–H bond [*Δ* ν (C–H)] was calculated. The last column in Table 1 shows the values of two shifts with respect to each phenyl ring for each local minimum. The calculated *Δ ν* (C–H) stretching vibration shifts are very small and upshifted (see Table 1). The blue-shift of the *ν* (C–H) stretching vibration is a known feature of C–H⋯π intermolecular hydrogen bonds and has been detected both in experimental and theoretical studies [30].

One of the methods used to confirm the formation of the hydrogen bond is AIM analysis [31]. There are general topological criteria for the existence of the hydrogen bond, namely the existence of the bond path between the proton donor and acceptor and the specified range of both the electron density [*ρ*(*r*)_BCP_] and its Laplacian [*Δ ρ*(*r*)_BCP_] at the bond critical point (BCP) [32]. No bond path between the proton donor and acceptor, or BCPs was found by AIM analysis for any of the local minima. Therefore, the interaction between the H atom of the C–H group and the π electron density cannot be called a H-bond according to the necessary conditions as given in [32]. Thus, none of the local minima shown in Fig. 2 reveal the true C–H⋯π hydrogen bond character as it was predicted for example for the model system of water and benzene molecules [33].

### Car-Parrinello molecular dynamics simulations

The results of DFT calculations suggest the possibility of phenyl ring rotation so, as the next step in the conformational analysis, CP-MD simulations were performed. All CP-MD runs mimic the dynamic flexibility of the 4,4′-MDI molecule and give insight into the structural rearrangements at finite temperature. The RT value under such conditions is close to 3 kJ mol^−1^ and, in comparison to the results of DFT calculations, should be high enough to overcome the energy barriers. The production run was 24 ps long, and indeed huge structural rearrangements could be observed.

**Fig. 3:**
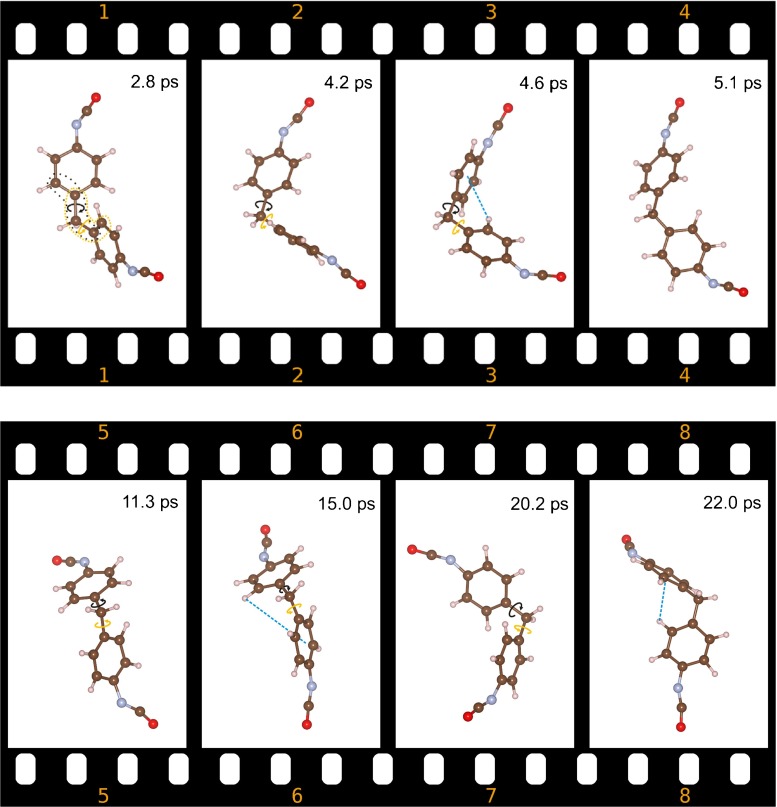
Time evolution of the structure of the 4,4′-MDI molecule from Car-Parrinello molecular dynamics (CP-MD) simulation. Each snapshot is presented with its time-stamp. The first four snapshots show one full simultaneous counter-rotation of both phenyl rings.* Black* and* orange arrows* around the C–C bonds show the direction of motion for the top and the bottom phenyl ring, respectively. Snapshots 5 and 7 show the intermediate structures in further rotations, whereas 6 and 8 refer to the equilibrium state. The presence of the C–H⋯π H-bond is marked by a* dashed line* where applicable

Figure 3 shows the most important steps in the CP-MD trajectory depicted as two rows of snapshots with the time stamps indicated. Successive snapshots were taken from the same perspective to observe, in the more transparent way possible, the motion of the phenyl rings. The first snapshot refers to the beginning of the phenyl ring rotation process and shows the structure of the 4,4′-MDI molecule at the time of 2.8 ps. Analysis of the structural rearrangements is based mainly on phenyl ring rotation so it is important to introduce some useful on-going definitions to describe this process. We consider clockwise and counter clockwise rotation of the phenyl ring, if looking at the N atom aligned in one axis with two C atoms, namely one C atom from the phenyl ring and the second C atom from the –CH_2_ group. The second snapshot was recorded at 4.2 ps; mutual rotation of both phenyl rings was observed. The phenyl ring at the top part of the second snapshot rotates clockwise whereas the bottom one rotates counter clockwise. Successive snapshots recorded at 4.6 and 5.1 ps show one full rotation of both phenyl rings.

**Fig. 4:**
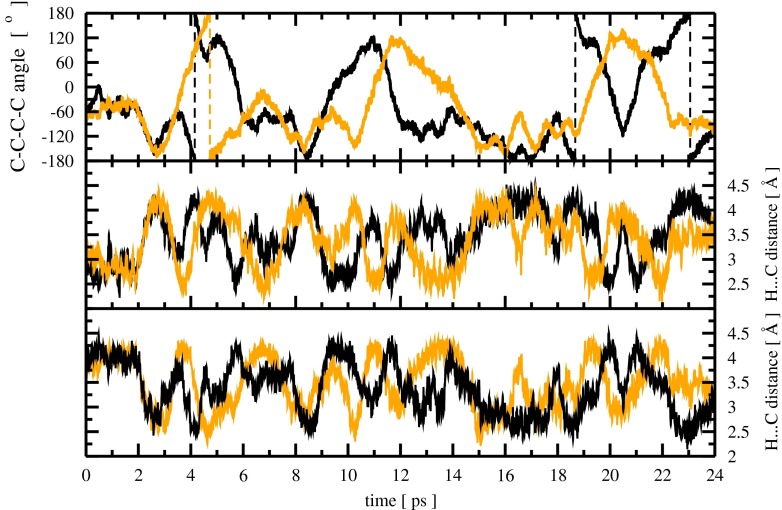
Time evolution of the two C–C–C–C dihedral angles (in degrees,* top*) and of the H⋯C distances (in Ångstroms,* bottom*) in the 4,4′-MDI molecule, obtained from a CP-MD run. The* black and orange line* refers to the different phenyl rings of the 4,4′-MDI molecule

The structural rearrangement can also be measured by two different dihedral angles depicted in the first snapshot in Fig. 3; the time evolution of both is shown in the top panel of Fig. 4. The black and orange line corresponds to the time evolution of two phenyl rings, namely the top and the bottom one of the 4,4′-MDI molecule from the first snapshot in Fig. 3. The middle and bottom panel in Fig. 4 show the time evolution of the four H⋯C distances between the hydrogen atom from one phenyl ring and the closest carbon atom from the second ring since at each phenyl ring two C–H bonds might be involved in the interaction with π electron density. One could plot all of four H⋯C distances at once but we split them into the two panels (middle and bottom) to make a better comparison with the time evolution of the dihedral angles from the top panel. The black line refers to both H H⋯C distances, where two hydrogen atoms belong to the top ring of the 4,4′-MDI molecule shown in the first snapshot of Fig. 3. The second line (orange) refers to the H⋯C distances between the C–H bonds from the bottom ring. The time evolution of all four H H⋯C distances is very complex and is thus not subject to detailed analysis here. The H⋯C distances may be compared with the corresponding ones obtained in the DFT calculations, presented in Table 1. The shortest H⋯C distance of 2.68 Å was observed for structure D; however the average H⋯C distance calculated for all local minima is 2.85 Å. The bottom panel in Fig. 4 shows that the structural rearrangements of the 4,4′-MDI molecule, namely the rotation of the phenyl rings, implies much shorter H⋯C distances in comparison to the data calculated for the local minima. Full rotation of the phenyl rings shown in the first four snapshots in Fig. 3 takes approximately 3 ps if we assume a starting point at 3.3 ps, which corresponds to the equilibrium value of the C–C–C–C dihedral angle predicted in the DFT calculations (see Fig. 4). The dashed lines in Fig. 4 show the full rotation of the phenyl rings. At the time of this counter rotation of the phenyl rings, very short H⋯C distances were detected (bottom panel in Fig. 4). The analysis of several snapshots with such short H⋯C distances shows that only one C–H⋯π contact is formed, and the phenyl rings are indeed perpendicular to each other. We checked the structures from two different snapshots taken at 5.7 and 9.3 ps to confirm if they reveal C–H⋯π hydrogen bonds based on AIM analysis. Contrary to the local minima, all the structures taken from the CP-MD run reveal a weak C–H⋯π H-bond according to the criteria given in [17]. In both structures, the bond path was detected between the donor and acceptor. Values of electron density [*ρ*(*r*)_BCP_] and its Laplacian [Δ ρ(*r*)_BCP_] equal to 0.013, 0.015 and 0.052, 0.049 a.u. are within the defined ranges of 0.002–0.035 and 0.024–0.139 a.u., respectively. On the basis of the time evolution of the H⋯C distances, the lifetime of such an intermediate structure with only one C–H⋯π might be estimated to be about 0.5 ps.

On the one hand, the formation of the C–H⋯π H-bond stabilizes the 4,4′-MDI molecule but on the other hand steric effects, which are connected with the perpendicular arrangement of the phenyl rings, perturb the equilibrium of the system. At the time of rotation, only one C–H⋯π H-bond is formed, whereas in the equilibrium the two weaker C–H⋯π contacts exist. AIM analysis does not predict a hydrogen bond for the local minima structures therefore we refer to them here as C–H⋯π contacts.

Analysis of a further part of the trajectory provided more detail concerning phenyl ring rotation. We cannot depict all of the structures in Fig. 3, which serves only as an illustration of the most important structural changes in the molecule. Snapshot 5 in Fig. 3 refers to the time of 11.3 ps, at which the next rotation has already started. At 9 ps, both phenyl rings start to counter rotate; however, at 10 ps one of them changes the direction of rotation (see Fig. 4). From this moment (10 ps) on, both phenyl rings rotate in one direction, namely anticlockwise, but not in the same phase. The time evolution of both C–C–C–C dihedral angles shows that, at 11 and 12 ps, the top and bottom phenyl rings, respectively, start to return to their initial positions. This strong structural rearrangement might be termed a half rotation because no turning point was observed. The 4,4′-MDI molecule comes to its equilibrium geometry at 15 ps (see snapshot number 6 in Fig. 3). The motion of both phenyl rings in the same direction does not lead to a full rotation.

The last part of the trajectory confirms that a successful process of full rotation calls for the counter rotation of both rings at the starting phase. Starting from the beginning of the 18th ps, both rings counter rotate exactly in the same manner, just as they do at 3.5 ps. However, the structural rearrangements are not exactly the same as those observed in the time region between 3 and 6 ps. Herein, at 20.2 ps (see snapshot 7 in Fig. 3), one ring changes the direction of rotation and returns to the equilibrium position at 22 ps (snapshot 8 in Fig. 3). The second phenyl ring continues clockwise rotation until one full rotation at 20 ps, then it starts a counter-clockwise rotation, reaching the equilibrium structure at 24 ps. In the time period between 18 and 24 ps, the bottom phenyl ring makes a half rotation whereas the top one makes two full rotations (see two dashed lines in Fig. 4). The time needed for one full rotation might then be estimated to be about 3 ps, as in the previous case.

Apart from the significant structural rearrangements of both phenyl rings, the isocyanate groups do not change their position much. Most of the time all the atoms in the NCO group are within the plane formed by the phenyl ring. Any out-of-plane position of atoms from the NCO group was monitored by the time evolution of the C–C–N–C dihedral angle. Oscillations from the equilibrium state sometimes even reach 60° but the average value is close to the in-plane position. After the full simulation time several half and full rotations were observed. The average time calculated for the isocyanate group to rotate about 180° was about 0.5 ps. Such rotation of the NCO group was much faster compared to rotation of the phenyl rings.

## Conclusions

The equilibrium structure of the 4,4′-MDI molecule was obtained from DFT calculations. The global minimum structure has two very weak interactions of the C–H⋯π type, which refer to the skewed arrangement of the phenyl rings with respect to each other. Additionally, this weak interaction was not classified as a hydrogen bond in AIM analysis based on commonly known criteria.

The conformational changes in the 4,4′-MDI molecule at finite temperature were observed in CP-MD simulations. Firstly, rotation of the phenyl rings was analyzed. The counter rotation of both rings increases the probability of full rotation, whereas rotation in the same phase restricts the motion of the phenyl rings to the half rotation case. The time needed for full rotation was estimated to be about 3 ps. AIM analysis confirmed the formation of one weak C–H⋯π H-bond in the intermediate structures with a lifetime of about 0.5 ps. Secondly, the motion of the isocyanate group was analyzed. All atoms of the NCO group oscillate with respect to their equilibrium position, namely in an in-plane position towards the phenyl ring. Several very fast rotations of the isocyanate group of about 180° were observed, with an average lifetime of 0.5 ps.
